# Novel Risk Score Incorporating Type-IV Collagen, Albumin, and Prothrombin Time (CAP score) to Predict 180-Day Surgery-Related Mortality After Liver Resection for Hepatocellular Carcinoma

**DOI:** 10.1245/s10434-025-17658-2

**Published:** 2025-06-23

**Authors:** Tomoaki Hayakawa, Shotaro Miyashita, Maiko Niki, Genki Tanaka, Takayuki Shimizu, Takamune Yamaguchi, Kyung-Hwa Park, Takatsugu Matsumoto, Takayuki Shiraki, Shozo Mori, Taku Aoki

**Affiliations:** Department of Hepato-Biliary-Pancreatic Surgery, Dokkyo Medical University, Tochigi, Japan

**Keywords:** Hepatocellular carcinoma, Liver resection, Surgery-related death, Albumin, Prothrombin time-international normalized ratio, Type IV collagen

## Abstract

**Background:**

Accurate preoperative risk assessment is crucial for patients undergoing liver resection for hepatocellular carcinoma (HCC). The present study developed and validated a novel scoring system for predicting 180-day surgery-related mortality.

**Patients and Methods:**

This retrospective cohort study enrolled patients who underwent liver resection for HCC between 2000 and 2024. The cohort was divided into training and validation sets on the basis of the operation dates. Multivariate analysis was performed to identify the independent predictors of 180-day surgery-related mortality. The resulting score was compared with the existing models.

**Results:**

Three independent predictors were identified and assigned one point each: type-IV collagen ≥ 7.5 ng/mL (odds ratio [OR]: 2.92; 95% confidence interval [CI] 1.20–7.65; *P* = 0.017), albumin (Alb) ≤ 3.4 g/dL (OR: 3.06, 95% CI 1.23–8.39; *P* = 0.016), and prothrombin time-international normalized ratio (PT-INR) ≥ 1.26 (OR: 2.82; 95% CI 1.14–6.70; *P* = 0.026). The 180-day surgery-related mortality rates for the low- (0 point), intermediate- (1–2 points), and high-risk (3 points) groups were 0.8%, 7.6%, and 26.7%, respectively, in the training cohort, and 1.7%, 6.5%, and 20.7%, respectively, in the validation cohort. The collagen–Alb–PT-INR (CAP) score demonstrated superior predictive performance (area under the curve [AUC]: 0.728) as compared with the stratified Model for End-Stage Liver Disease score (AUC: 0.557, *P* < 0.001), the Child–Pugh classification (AUC: 0.637, *P* < 0.001), and the Alb–bilirubin grade (AUC: 0.668, *P* = 0.014).

**Conclusions:**

The CAP score is a simple and effective tool for predicting 180-day surgery-related mortality post-liver resection for HCC.

**Supplementary Information:**

The online version contains supplementary material available at 10.1245/s10434-025-17658-2.

Hepatocellular carcinoma (HCC) remains one of the leading causes of cancer-related deaths worldwide, with surgical resection being a crucial curative treatment option for eligible patients.^1,2^ Despite the advances in surgical techniques and perioperative management, postoperative mortality after liver resection for HCC remains a considerable concern, with reported mortality rates of approximately 1–3% in recent years.^3–5^ Accurate preoperative risk assessment is essential for optimal patient selection and informed decision-making.^6^

Although various mortality prediction models exist, those models utilizing postoperative data cannot be applied preoperatively, limiting their usefulness in surgical decision-making.^4,7,8^ Although the Model for End-Stage Liver Disease (MELD) score,^9^ Child–Pugh classification,^10^ and albumin (Alb)–bilirubin (ALBI) grade are commonly used preoperatively for assessing liver function,^11^ they may have limitations in predicting postoperative mortality following HCC resection. The MELD score, primarily developed for liver transplantation of end-stage liver disease, may not adequately capture the acute complications of liver resection. Child–Pugh classification, which includes subjective assessments such as ascites and encephalopathy, can be less accurate in patients with relatively preserved liver function, such as those with early-stage HCC. The ALBI grade, designed for long-term prognosis, may not be optimal for predicting early postoperative mortality. The scoring systems that incorporate factors such as tumor characteristics (e.g., tumor size, number, and location) and the extent of hepatectomy (e.g., major or minor hepatectomy) often require specialized assessments by hepatobiliary surgeons or radiologists, which can be challenging for physicians outside of these specialties.^12,13^

Recent studies have highlighted the potential role of novel biomarkers, particularly type-IV collagen—a basement membrane protein that accumulates during the progression of liver fibrosis—in predicting surgical outcomes.^14–16^ In addition to the novel fibrosis marker, conventional laboratory parameters, including Alb,^17–19^ a liver-synthesized protein reflecting nutritional status and hepatic function, and prothrombin time-international normalized ratio (PT-INR), a standardized coagulation test for determining liver synthetic capacity,^11,20,21^ have also shown promise in risk stratification. However, no existing scoring system effectively combines these complementary parameters for predicting post-hepatectomy mortality.

The present study aimed to develop and validate a novel, simple, objective, and clinically applicable risk score to predict 180-day surgery-related mortality following liver resection for HCC. This score incorporates the preoperative nutritional status (Alb), hepatic reserve (PT-INR), and liver fibrosis (type-IV collagen) and is designed for use by physicians, regardless of specialty.

## Patients and Methods

### Patients

This retrospective cohort study enrolled patients who underwent liver resection for HCC at Dokkyo Medical University between 2000 and 2024. To minimize selection bias, the cohort was divided into training and validation sets on the basis of the odd- and even-numbered operation dates. This allocation method was chosen to ensure a balanced distribution of patients from all periods across both cohorts, thereby minimizing potential bias from temporal changes in surgical techniques and perioperative management throughout the study period. The patients’ clinical and demographic characteristics were collected from a computerized database and analyzed retrospectively with approval from the institutional review board (approval number: R-14-11J). Informed consent was obtained from the patients using an opt-out approach on our institutional website. The present study followed the principles outlined in the Declaration of Helsinki.

### Surgical Procedures

The patients and operative procedures were selected based on the liver function parameters, including serum bilirubin levels, presence of uncontrolled ascites, and indocyanine green retention rate at 15 min (ICG-R15), regardless of whether the patients had an underlying cirrhosis or portal hypertension. Following our institutional criteria for patients with normal bilirubin levels, we performed enucleation for those with an ICG-R15 of > 40%, limited resection for those with an ICG-R15 of < 40%, subsegmentectomy or monosegmentectomy for those with an ICG-R15 of < 30%, left hepatectomy or sectionectomy for those with an ICG-R15 of < 20%, and right hepatectomy or resection of three sections for those with an ICG-R15 of < 10%. These selection criteria remained consistent throughout the study period. Parenchymal transection was performed using either the clamp crushing method or the cavitron ultrasonic surgical aspirator with the intermittent Pringle’s maneuver. All procedures were performed either through a J-shaped or an inverted L-shaped laparotomy incision for open surgery or using three to six ports for laparoscopic procedures. Drainage tube placement was determined at the discretion of the attending surgeons. The complexity of liver resection was assessed using our previously developed modified three-level classification.^22–24^ The procedures were categorized as follows: grade I-single (single-wedge partial resection and left lateral sectionectomy), grade I-multiple (multiple-wedge partial resections), grade II (anterolateral segmentectomy and left hepatectomy), and grade III (posterosuperior segmentectomy, right posterior sectionectomy, right hepatectomy, central hepatectomy, and extended left/right hepatectomy) (Supplementary Fig. 1).

### Data Collection

The preoperative variables included patient demographics, comorbidities, laboratory data, and liver function parameters collected within 1 month before surgery. For almost all cases, the laboratory parameters included complete blood count, liver function tests, coagulation profiles, and specific biomarkers, such as type-IV collagen, which were obtained from tests performed during this 1-month preoperative period. Data on the surgical variables and postoperative outcomes were collected from the patients’ medical records. Deaths from liver failure and other causes were defined as surgery-related mortality, whereas cancer-related deaths and deaths from unknown causes were excluded from this definition and subsequent analyses. All deaths other than cancer-related deaths and deaths from unknown causes were included as surgery-related mortality, as we cannot definitively assert that they were unrelated to the surgical intervention. In our analysis, we evaluated mortality within 180 days after liver resection, rather than the more conventional 90-day postoperative period. This extended timeframe was selected to comprehensively capture both early and potential delayed complications that may be attributable to the surgical intervention and postoperative course. We recognized that late-onset mortality in patients with HCC might occur due to delayed liver failure,^25^ late infectious complications,^26^ and gradual functional decline—complications that may not be fully captured by the conventional 90-day mortality endpoint.

Among several available liver fibrosis markers, such as type III procollagen,^27^ hyaluronic acid,^28^ and M2BPGi,^29^ we selected type-IV collagen for this study on the basis of its strong correlation with hepatic fibrosis progression. While our institution also measured other fibrosis markers including hyaluronic acid and type III procollagen, we excluded type III procollagen from analysis owing to measurement method changes during the study period resulting in incompatible data. Hyaluronic acid showed multicollinearity with type-IV collagen in our preliminary statistical assessment, leading to its exclusion from the multivariate model. M2BPGi was not routinely measured at our institution during the study period.

### Risk Score Development and Validation

On the basis of the identified independent risk factors from the multivariate analysis, we planned to develop a scoring system by assigning points to each significant factor. The weight of the point assignment was determined on the basis of each factor’s relative strength of association with 180-day surgery-related mortality. The patients were then stratified into risk groups according to their total score, with the cutoff values for risk stratification determined by the distribution of surgery-related mortality rates across different score values in the training cohort.

The predictive performance of the scoring system was evaluated by performing an area under the receiver operating characteristic curve (AUC) analysis and compared with the existing liver function assessment tools (ALBI grade [categorized as grade 1: ≤ −2.60, grade 2: > −2.60 to ≤ −1.39, and grade 3: >−1.39],^11^ MELD score [categorized as < 10: low risk, 10–19: moderate risk, 20–29: high risk, and ≥ 30: very high risk],^9,30^ and Child–Pugh classification [classes A, B, and C]).^10^ All statistical analyses were performed using JMP 18.0.1 (SAS Institute Inc., Cary, NC, USA), and *P* < 0.05 was considered statistically significant.

## Results

### Patient Characteristics

Among the 1200 consecutive patients who underwent liver resection for HCC, 1197 were included in the final analysis after excluding three patients who underwent liver transplantation (Supplementary Fig. 2). All patients except one were Japanese. The cohort was then divided into the training (*n* = 623) and validation (*n* = 574) sets on the basis of the operation dates. The patients’ median age was 69 years (interquartile range [IQR]: 63–74 years), and 79.7% were male. The majority of the patients had hepatitis B or C virus infection (78.9%), and a significant proportion had comorbidities, such as hypertension (76.0%) and diabetes mellitus (52.4%). Most patients (84.2%) had Child–Pugh grade A liver function, with a median MELD score of 7.7 (IQR: 7.0–8.7). Preoperative liver function tests showed a median Alb level of 3.6 g/dL (IQR: 3.2–4.0 g/dL), total bilirubin level of 0.6 mg/dL (IQR: 0.5–0.8 mg/dL), PT-INR of 1.10 (IQR: 1.03–1.18), and type-IV collagen level of 6.2 ng/mL (IQR: 4.9–8.6 ng/mL) (Table 1).

**Table 1:** Demographic background and clinical characteristics of patients who underwent liver resection for hepatocellular carcinoma (*N* = 1197)

Characteristic	Total cohort (*N* = 1197)	Training cohort (*N* = 623)	Validation cohort (*N* = 574)	*P*
*Patient factors*				
Age, years	69 (63–74)	69 (63–74)	70 (64–75)	0.026
Sex, *n* (%)				
Male	952 (79.7)	488 (78.5)	464 (81.0)	0.279
Female	243 (20.3)	134 (21.5)	109 (19.0)	
BMI, kg/m^2^	23.1 (20.6–25.7)	23.3 (20.6–25.9)	23.0 (20.5–25.4)	0.352
ASA-PS ≥ 3, *n* (%)	43 (7.3)	19 (6.1)	24 (8.9)	0.196
ICG, %	14 (9–20)	13 (9–20)	14 (9–21)	0.187
Child–Pugh grade A: B: C, *n* (%)	1007 (84.2):188 (15.7):1 (0.1)	539 (86.7): 83 (13.3): 0 (0)	468 (81.5): 105 (18.3): 1 (0.2)	0.030
MELD	7.7 (7.0–8.7)	7.7 (6.9–8.6)	7.7 (7.0–8.8)	0.366
Initial hepatectomy, *n* (%)	896 (74.9)	470 (75.6)	426 (74.2)	0.592
Background viral status				
HBV, *n* (%)	262 (21.9)	131 (21.0)	131 (22.8)	0.453
HCV, *n* (%)	340 (28.4)	183 (29.4)	157 (27.4)	0.438
HBV + HCV, *n* (%)	332 (28.3)	166 (26.7)	167 (29.1)	0.345
NBNC, *n* (%)	256 (21.4)	140 (22.5)	116 (20.2)	0.340
Comorbidities				
Diabetes mellitus, *n* (%)	428 (52.4)	218 (51.1)	210 (53.9)	0.425
Hypertension, *n* (%)	642 (76.0)	336 (76.2)	306 (75.7)	0.880
Cardiovascular diseases, *n* (%)	61 (10.5)	30 (9.9)	31 (11.2)	0.603
Cerebrovascular diseases, *n* (%)	75 (12.5)	41 (13.1)	34 (12.0)	0.689
Blood test				
White blood cell count,/μL	4800 (3900–6000)	4900 (3900–6030)	4800 (4000–5900)	0.428
Neutrophil count, %	2730 (2000–3691)	2750 (1990–3791)	2670 (2035–3604)	0.595
Lymphocyte count, %	1408 (1061–1835)	1442 (1076–1848)	1373 (1039–1831)	0.431
Platelet, × 10^4^/μL	14.5 (10.4–19.3)	14.6 (10.1–19.3)	14.5 (10.7–19.4)	0.989
Aspartate transaminase, IU/L	34 (25–47)	34 (25–45)	35 (26–48)	0.170
Alanine transaminase, IU/L	28 (18–43)	28 (18–43)	29 (19–43)	0.505
C-reactive protein, mg/dL	0.17 (0.10–0.30)	0.16 (0.10–0.30)	0.19 (0.10–0.30)	0.284
Albumin, g/dL	3.6 (3.2–4.0)	3.7 (3.2–4.0)	3.6 (3.2–4.0)	0.204
Total bilirubin, mg/dL	0.6 (0.5–0.8)	0.6 (0.5–0.8)	0.6 (0.5–0.8)	0.356
PT-INR	1.10 (1.03–1.18)	1.10 (1.03–1.17)	1.10 (1.03–1.19)	0.638
Hyaluronic acid, ng/mL	119 (64–243)	109 (61–234)	125 (65–250)	0.230
Type IV collagen, ng/mL	6.2 (4.9–8.6)	6.3 (4.8–8.6)	6.2 (4.9–8.7)	0.883

Values are expressed as *n* (%) or median (interquartile range)

ASA-PS, American Society of Anesthesiologists-physical status; BMI, body mass index; HBV, hepatitis B virus; HCV, hepatitis C virus; ICG, indocyanine green retention rate; MELD, Model for End-Stage Liver Disease; NBNC, non-B non-C hepatitis; PT-INR, prothrombin time-international normalized ratio

### Surgical Characteristics and Outcomes

The majority of surgeries (95.9%) were performed via the open approach, with a median operative time of 289 min (IQR: 223–365 min) and estimated blood loss of 500 mL (IQR: 274–924 mL). On the basis of our modified three-level complexity classification, the procedures were classified as follows: grade I-single (34.5%), grade I-multiple (17.5%), grade II (13.1%), and grade III (34.6%). According to the TNM classification, 226 (19.1%), 473 (40.0%), 357 (30.2%), and 127 (10.7%) patients had stage I, II, III, and IV diseases, respectively. The postoperative complications included bile leakage (10.8%), intra-abdominal abscess (6.2%), and liver failure (4.2%). Among the 1197 patients, 84 (7.0%) died within 180 days postoperatively, comprising 60 patients (5.0%) who had surgery-related deaths (34 patients [2.8%] from liver failure and 26 patients [2.2%] from other causes), 17 patients (1.6%) who had cancer-related deaths, and 7 patients (0.6%) who died from unknown causes (Table 2). Detailed information regarding the causes of death and survival duration for all 60 surgery-related mortality cases is presented in Supplementary Table 1. This data illustrates the temporal distribution of mortality, revealing that 35 patients (58.3%) died within the first 90 days after surgery, while 25 patients (41.7%) died between days 91 and 180.

**Table 2:** Surgical and postoperative outcomes in patients undergoing liver resection (*N* = 1197)

Characteristic	Total cohort (*N* = 1197)	Training cohort (*N* = 623)	Validation cohort (*N* = 574)	*P*
Surgical outcomes				
Operating time, min	289 (223–365)	289 (228–361)	289 (222–369)	0.818
Estimated blood loss, mL	500 (274–924)	500 (272–950)	500 (275–897)	0.562
Approaches, *n* (%)				
Open	1148 (95.9)	600 (96.3)	548 (95.5)	0.465
Minimally invasive surgery	49 (4.1)	23 (3.7)	26 (4.5)	
Complexity classification, *n* (%)*				
Grade I-single	406 (34.5)	200 (32.1)	220 (38.3)	0.142
Grade I-multiple	206 (17.5)	109 (17.5)	97 (16.9)	
Grade II	154 (13.1)	88 (14.1)	69 (12.0)	
Grade III	407 (34.6)	226 (36.3)	188 (32.8)	
TNM classification				
Stage I	226 (19.1)	114 (18.5)	112 (19.8)	0.392
Stage II	473 (40.0)	261 (42.3)	212 (37.5)	
Stage III	357 (30.2)	180 (29.2)	177 (31.3)	
Stage IV	127 (10.7)	62 (10.1)	65 (11.5)	
Postoperative outcomes, *n* (%)				
Liver failure	35 (4.2)	14 (3.2)	21 (5.4)	0.130
Postoperative bleeding	17 (2.1)	11 (2.5)	6 (1.6)	0.331
Bile leakage	126 (10.8)	65 (10.6)	61 (10.9)	0.473
Intraabdominal abscess	51 (6.2)	27 (6.2)	24 (6.2)	0.967
Clavien–Dindo grade ≥ II	477 (40.3)	237 (38.5)	240 (42.3)	0.193
90-day mortality	44 (3.7)	25 (4.0)	19 (3.3)	0.518
90-day surgery-related death	35 (2.9)	20 (3.2)	15 (2.6)	0.537
180-day mortality	84 (7.0)	44 (7.1)	40 (7.0)	0.949
180-day surgery-related death	60 (5.0)	32 (5.1)	28 (4.9)	0.838
Liver failure	34 (2.8)	18 (2.9)	16 (2.8)	0.916
Others	26 (2.2)	14 (2.3)	12 (2.1)	0.853
180-day non-surgery-related death	24 (2.0)	12 (1.9)	12 (2.1)	0.839
Cancer death	17 (1.4)	9 (1.4)	8 (1.4)	0.941
Unknown	7 (0.6)	3 (0.5)	4 (0.7)	0.625

Values are expressed as *n* (%) or median (interquartile range)

*Modified three-level complexity classification. Grade I-single (single wedge resection and left lateral sectionectomy), grade I-multiple (multiple wedges resections), grade II (anterolateral segmentectomy and left hepatectomy), and grade III (posterosuperior segmentectomy, right posterior sectionectomy, right hepatectomy, central hepatectomy, and extended left/right hepatectomy)

### Risk Factors for 180-Day Surgery-Related Mortality

In the training cohort (*n* = 623), univariate analysis identified five variables significantly associated with 180-day surgery-related mortality, which were as follows: Alb level of ≤ 3.4 g/dL (odds ratio [OR]: 6.63, 95% confidence interval [CI]: 2.97–16.8, *P* < 0.001), PT-INR of ≥ 1.26 (OR: 5.09, 95% CI 2.26–10.8, *P* < 0.001), and type-IV collagen level of ≥ 7.5 ng/mL (OR: 5.31, 95% CI 2.37–13.0, *P* < 0.001). After the multivariate analysis, the following three independent predictors remained significant: Alb level of ≤ 3.4 g/dL (OR: 3.06, 95% CI 1.23–8.39, *P* = 0.016), PT-INR of ≥ 1.26 (OR: 2.82, 95% CI 1.14–6.70, *P* = 0.026), and type-IV collagen level of ≥ 7.5 ng/mL (OR: 2.92, 95% CI 1.20–7.65, *P* = 0.017) (Table 3). In addition to the markers included in the final model, we also analyzed hyaluronic acid as a potential fibrosis marker, which showed a significant association with 180-day surgery-related mortality in univariate analysis (OR: 4.99, 95% CI 1.93–15.5, *P* < 0.001; Supplementary Table 2). However, owing to multicollinearity with type-IV collagen and its lower predictive strength in our preliminary analyses, hyaluronic acid was excluded from the multivariate model in favor of type-IV collagen.

**Table 3:** Uni- and multivariate analyses for 180-day surgery related mortality in training cohort (*N* = 623)

Factor	Univariate analysis			Multivariate analysis		
	OR	95% CI	*P*	OR	95% CI	*P*
Age, year ≥72	2.02	0.98–4.14	0.056			
Sex; male versus female	0.43	0.51–0.94	0.035			
BMI kg/m^2^ ≥ 19.1	0.63	0.25–1.94	0.390			
ASA-PS ≥ 3	0.76	0.04–4.01	0.791			
Background status						
HBV/HCV hepatitis	1.61	0.66–4.82	0.315			
Diabetes mellitus	0.78	0.31–1.91	0.579			
Hypertension	1.13	0.44–3.50	0.809			
Approaches						
Open versus minimally invasive surgery	–	–	0.116			
Procedure complexity classification*						
Grade I-single	Reference					
Grade I-multiple	1.29	0.52–3.11	0.570			
Grade II	0.17	0.01–0.85	0.028			
Grade III	0.60	0.24–1.41	0.241			
Liver function^†^						
ICG-R15 ≥ 20%	2.05	0.97–4.23	0.060			
Albumin ≤ 3.4 g/dL	6.63	2.97–16.8	< 0.001	3.06	1.23–8.39	0.016
Total bilirubin ≥ 2.0 mg/dL	–	–	0.515			
PT-INR ≥ 1.26	5.09	2.26–10.8	< 0.001	2.82	1.14–6.70	0.026
Type IV Collagen ≥ 7.5 ng/mL	5.31	2.37–13.0	< 0.001	2.92	1.20–7.65	0.017

*Modified three-level complexity classification. Grade I-single (single wedge partial resection and left lateral sectionectomy), grade I-multiple (multiple wedges partial resections), grade II (anterolateral segmentectomy and left hepatectomy), and grade III (posterosuperior segmentectomy, right posterior sectionectomy, right hepatectomy, central hepatectomy, and extended left/right hepatectomy)

^†^Cut-off values were determined using receiver operating characteristic curve analysis

The multivariate analysis was performed using backward stepwise selection with variables that were significant (*P* < 0.05) in univariate analysis. Variables were eliminated from the model if *P* > 0.05

ASA-PS, American society of Anesthesiologists-physical status; BMI, body mass index; CI, confidence intervals; HBV, hepatitis B virus; HCV, hepatitis C virus; ICG-R15, indocyanine green retention rate at 15 minutes; OR, odds ratio; PT-INR, prothrombin time-international normalized ratio

### Development and Application of the Risk Classification System

**Fig. 1:**
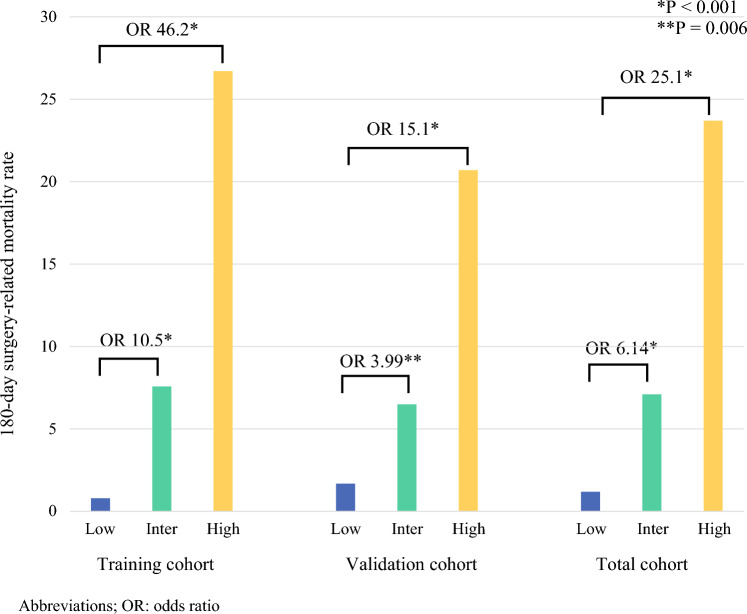
Comparison of 180-day surgery-related mortality rates and odds ratios among the risk groups (low-, intermediate-, and high-risk groups) in the training, validation, and total cohorts based on the collagen–Alb–PT-INR classification system

Given that all identified factors showed similar ORs of approximately 3 (Alb ≤ 3.4 g/dL, OR 3.06; PT-INR ≥ 1.26, OR 2.82; and type-IV collagen score ≥ 7.5 ng/mL, OR 2.92), we assigned one point to each factor. On the basis of these three independent predictors, the patients were classified into the following three risk groups: low- (0 point), intermediate- (1–2 points), and high-risk (3 points) groups. In the training cohort (*n* = 623), the distribution of patients across the risk groups was as follows: low- (*n* = 256, 45.6%), intermediate- (*n* = 275, 49.0%), and high-risk (*n* = 30, 5.3%) groups. Owing to the missing data on the preoperative parameters, 62 patients could not be classified into the risk groups; hence, they were excluded from the analysis. The 180-day surgery-related mortality rates were 0.8%, 7.6%, and 26.7% for the low-, intermediate-, and high-risk groups, respectively. The intermediate-risk group showed a significantly higher risk than the low-risk group (OR: 10.5, 95% CI 3.04–66.1, *P* < 0.001). Similarly, the high-risk group demonstrated an even greater increase in risk than the low-risk group (OR: 46.2, 95% CI 10.8–319, *P* < 0.001; Table 4). The progressive increase in the mortality rates across the risk groups and the corresponding odds ratios demonstrate the robust stratification capability of our scoring system (Fig. 1).

**Table 4:** Predictive performance of Alb-PT-INR-collagen score for 180-day surgery-related mortality

(A) Training cohort (*N* = 623)				
*Risk group	*n*, (%)	180-day mortality, *n* (%)	OR (95%CI)	*P*
Low (0)	256 (45.6)	2 (0.8)	Reference	
Intermediate (1–2)	275 (49.0)	21 (7.6)	10.5 (3.04–66.1)	< 0.001
High (3)	30 (5.3)	8 (26.7)	46.2 (10.8–319)	< 0.001
(B) Validation cohort (*N* = 574)				
*Risk group	*n*, (%)	180-day mortality, *n* (%)	OR (95% CI)	*P*
Low (0)	235 (44.6)	4 (1.7)	Reference	
Intermediate (1–2)	263 (49.9)	17 (6.5)	3.99 (1.45–14.0)	0.006
High (3)	29 (5.5)	6 (20.7)	15.1 (4.02–62.7)	< 0.001
(C) Total cohort (*N* = 1197)				
*Risk group	*n*, (%)	180-day mortality, *n* (%)	OR (95% CI)	*P*
Low (0)	491 (45.1)	6 (1.2)	Reference	
Intermediate (1–2)	538 (49.4)	38 (7.1)	6.14 (2.57–14.7)	< 0.001
High (3)	59 (5.4)	14 (23.7)	25.1 (9.21–68.6)	< 0.001
(D) Between-group comparison in the validation cohort				
Comparison	OR (95% CI)	*P* value		
High (3) versus low (0)	15.1 (4.02–62.7)	< 0.001		
High (3) versus intermediate (1–2)	3.77 (1.26–10.1)	0.019		
Intermediate (1–2) versus low (0)	3.99 (1.45–14.0)	0.006		

OR, odds ratio; CI, confidence interval

*Risk classification based on three preoperative factors (1 point each): albumin ≤ 3.4 g/dL, PT-INR ≥ 1.26, and type IV collagen ≥ 7.5 ng/mL

*Patients with missing data for risk group classification were excluded from the analysis

### Validation of the Risk Classification System

In the validation cohort (*n* = 574), a similar distribution of risk groups was observed: low- (*n* = 235, 44.6%), intermediate- (*n* = 263, 49.9%), and high-risk (*n* = 29, 5.5%) groups. Due to the missing data, 47 patients could not be classified and were excluded from the analysis. The 180-day surgery-related mortality rates were 1.7%, 6.5%, and 20.7% for the low-, intermediate-, and high-risk groups, respectively. The risk stratification remained significant, with the intermediate-risk group showing a higher risk than the low-risk group (OR: 3.99, 95% CI 1.45–14.0, *P* = 0.006), and the high-risk group demonstrating the highest risk (OR: 15.1, 95% CI 4.02–62.7, *P* < 0.001). In addition, the high-risk group showed a significantly higher mortality rate than the intermediate-risk group (OR: 3.77, 95% CI 1.26–10.1, *P* = 0.019; Table 4). The consistency of risk stratification between the training and validation cohorts is demonstrated in Fig. 1, supporting the robust predictive value of our scoring system.

### Comparison of Our Scoring System with the Existing Risk Assessment Tools

**Fig. 2:**
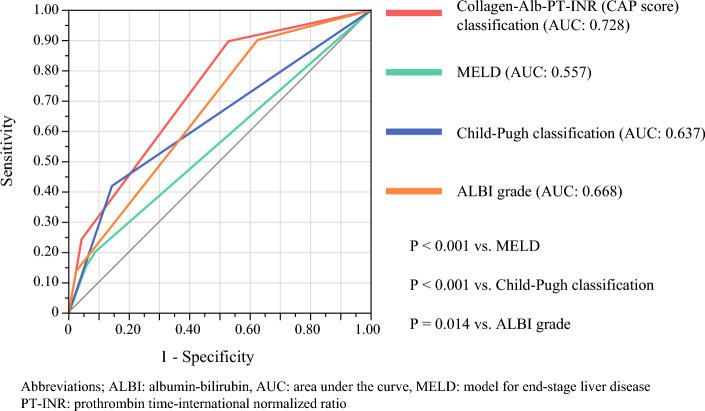
Comparison of receiver operating characteristic curves for predicting 180-day surgery-related mortality: collagen–Alb–PT-INR classification versus conventional liver function scoring systems (MELD, Child–Pugh, and ALBI)

In the total cohort, our classification system (AUC: 0.728) showed significantly better predictive performance than the stratified MELD score (AUC: 0.557, *P* < 0.001), Child–Pugh classification (AUC: 0.637, *P* < 0.001), and ALBI grade (AUC: 0.668, *P* = 0.014) (Fig. 2; Table 5). Similarly, we evaluated an alternative classification system using hyaluronic acid instead of type-IV collagen. This hyaluronic acid–Alb–PT-INR classification demonstrated slightly lower predictive performance (AUC: 0.721) compared with the CAP (AUC: 0.728) score and failed to show statistically significant superiority over the ALBI grade (*P* = 0.067; Supplementary Table 3).

**Table 5:** Comparison of models for 180-day surgery-related mortality using logistic regression and receiver operating characteristic curve analyses in total cohort

Models	180-day mortality	
	AUC	P*
Alb-PT-INR-collagen classification	0.728	–
MELD score	0.557	< 0.001
Child–Pugh classification	0.637	< 0.001
ALBI grade	0.668	0.014

ALBI, Albumin-Bilirubin; AUC, area under the curve; MELD, Model for End-Stage Liver Disease; PT-INR, prothrombin time-international normalized ratio

*Versus Alb-PT-INR-collagen classification model

## Discussion

In this retrospective cohort study involving 1197 patients undergoing liver resection for HCC, we developed and validated a novel preoperative risk-scoring system—the CAP score—based on the following three preoperative objective laboratory parameters: type-IV collagen ≥ 7.5 ng/mL, serum Alb ≤ 3.4 g/dL, and PT-INR ≥ 1.26. This novel scoring system effectively stratified the patients into groups with a low, intermediate, and high risk for 180-day surgery-related mortality.

Our scoring system comprises three components, each reflecting a different aspect of surgical risk. Serum Alb reflects liver synthetic function and indicates nutritional status, which plays a crucial role in the patients’ postoperative recovery through wound healing and immune response.^17–19^ A previous study has shown that preoperative hypoalbuminemia is strongly associated with increased rates of surgical site infections and delayed recovery, highlighting its importance as a modifiable risk factor.^31^ Elevated PT-INR reflects a decreased hepatic synthesis of coagulation factors, indicating impaired synthetic capacity and a higher risk of bleeding complications.^20,21,32^ Type-IV collagen, a component of the extracellular matrix, is a non-invasive marker of hepatic fibrosis severity and provides insight into the regenerative potential of the remaining liver tissue.^14–16,33^ While other fibrosis markers such as hyaluronic acid,^28^ type III procollagen,^27^ and M2BPGi^29^ have demonstrated utility in assessing liver fibrosis, our analysis identified type-IV collagen as the most predictive marker for postoperative outcomes in our cohort. Integrating these three parameters, the CAP score captures both the functional and structural aspects of liver health, which are crucial for achieving good surgical outcomes.

CAP score accurately predicted the 180-day surgery-related mortality (AUC: 0.728) using only three variables. Traditional scoring systems have limitations in predicting 180-day surgery-related mortality. The Child–Pugh score (AUC: 0.637) uses five variables, including subjective assessments such as encephalopathy and ascites, which may not be present in patients with early-stage HCC. Furthermore, owing to the poor outcomes associated with Child–Pugh class C liver disease, there has been a strong tendency to avoid surgery in this case, resulting in our cohort consisting almost entirely of patients with Child–Pugh class A or B liver disease, which limited effective stratification. The ALBI grade (AUC: 0.668), an objective and simplified assessment tool that uses two variables, was developed to predict the long-term outcomes of patients after HCC resection. However, its utility for predicting the 180-day outcomes remains unclear because it was not originally designed for short-term mortality prediction. The MELD score (AUC: 0.557) uses three variables and, while widely used for prioritizing liver transplant candidates, may not be optimally calibrated for surgical patients because it was primarily designed to assess patients with end-stage liver disease requiring transplantation rather than those eligible for hepatic resection. Our Alb–PT-INR–collagen scoring system, specifically designed for 180-day surgery-related mortality prediction, overcomes these limitations while maintaining simplicity and achieving better predictive performance.

The ability of the CAP score to stratify patients preoperatively into risk categories has considerable clinical implications. The high mortality rate in high-risk patients (3 points; 180-day surgery-related mortality: 26.7% in the training cohort) suggests that alternative treatments, including transarterial chemoembolization or radiofrequency ablation, should be carefully considered. However, when surgery is the only viable option for curative treatment, these high-risk patients should be managed with heightened precautions, including mandatory postoperative intensive care unit care and operation by attending surgeons with extensive hepatobiliary experience, to optimize the surgical outcomes, despite their elevated risk profile. Furthermore, although we cannot make definitive conclusions, owing to the limited number of minimally invasive surgery (MIS) cases in our cohort, previous studies have reported lower postoperative mortality rates with MIS compared with open surgery.^34,35^ This suggests that selecting MIS over open surgery, when technically feasible, might potentially reduce mortality rates in high-risk patients identified by the CAP score. This possibility warrants further investigation in cohorts with a higher proportion of MIS procedures.

For the intermediate-risk patients (1–2 points; 180-day surgery-related mortality: 7.6% in the training cohort), the scoring system helps guide perioperative management through more intensive postoperative monitoring and early intervention protocols. Although routine intensive care unit admission may not be necessary for all intermediate-risk patients, this group may benefit from a lower threshold for postoperative intensive care unit care when considering additional risk factors, such as performance status and comorbidities. In addition, the favorable outcomes in low-risk patients (0 point; 180-day surgery-related mortality: 0.8% in the training cohort) indicate that these cases could safely be assigned to less experienced surgeons as training opportunities. The simple three-point scoring system, calculated using data from routine laboratory tests, facilitates risk assessment across specialized hepatobiliary centers and general practice settings and enhances patient counseling by providing clear, evidence-based estimates of surgical risk.

Liver resection is increasingly transitioning from open to minimally invasive approaches at many institutions, and MIS is expected to become even more prevalent in hepatobiliary surgery. The CAP score was developed in a cohort where open surgery was the predominant approach (95.9%). However, we believe that the CAP score would maintain its predictive value in patients undergoing MIS since the components of the score—type-IV collagen, Alb, and PT-INR—reflect the patient’s liver fibrosis, synthetic function, and nutritional status, which are intrinsic patient factors independent of the surgical approach. Previous studies have suggested that MIS may be associated with lower postoperative mortality rates compared with open surgery,^34,35^ potentially due to reduced physiological stress and inflammatory response. This raises the possibility that MIS might be particularly beneficial for high-risk patients identified by the CAP score. However, the limited number of MIS cases in our cohort prevents us from making definitive recommendations regarding the optimal surgical approach for patients stratified by the CAP score.

Our study has several limitations. The single-center, retrospective design may affect the generalizability of the study findings. Moreover, the measurement of type-IV collagen lacks standardization across institutions. It should also be noted that two of our three variables (albumin and PT-INR) are related to liver synthetic function, which could theoretically affect their independence despite being identified as independent predictors in our multivariate analysis. These parameters might potentially become confounding factors when analyzed with larger datasets in future studies. In addition, the low proportion of MIS cases (4.1%) in our cohort may limit the applicability of CAP score to centers where MIS is the predominant approach. Whether the CAP score performs equally well in patients undergoing MIS requires further validation in cohorts with a higher representation of MIS. Future research should focus on multicenter validation and evaluating whether score-guided interventions improve patient outcomes. Moreover, studies exploring preoperative optimization strategies for high-risk patients could further enhance the clinical utility of this system.

## Conclusions

The CAP score greatly advances preoperative risk stratification for patients undergoing liver resection for HCC. This scoring system provides a more accurate prediction of 180-day surgery-related mortality compared with the existing models. Its simplicity and reliance on objective parameters make it a practical tool for guiding patient selection and preoperative management.

## Supplementary Information

Below is the link to the electronic supplementary material.

Supplementary Figure 1. Modified complexity classification [from our previous work (^22–24^)]

Supplementary Figure 2. Study flow diagram of patient selection and cohort allocation

Supplementary file3 (DOCX 16 kb)
