# Supplementary figures and images for: Novel Risk Score Incorporating Type-IV Collagen, Albumin, and Prothrombin Time (CAP score) to Predict 180-Day Surgery-Related Mortality After Liver Resection for Hepatocellular Carcinoma
Source: Ann Surg Oncol. 2025 Jun 23;32(10):7970–80. doi: 10.1245/s10434-025-17658-2 (PMC12454577; doi:10.1245/s10434-025-17658-2)

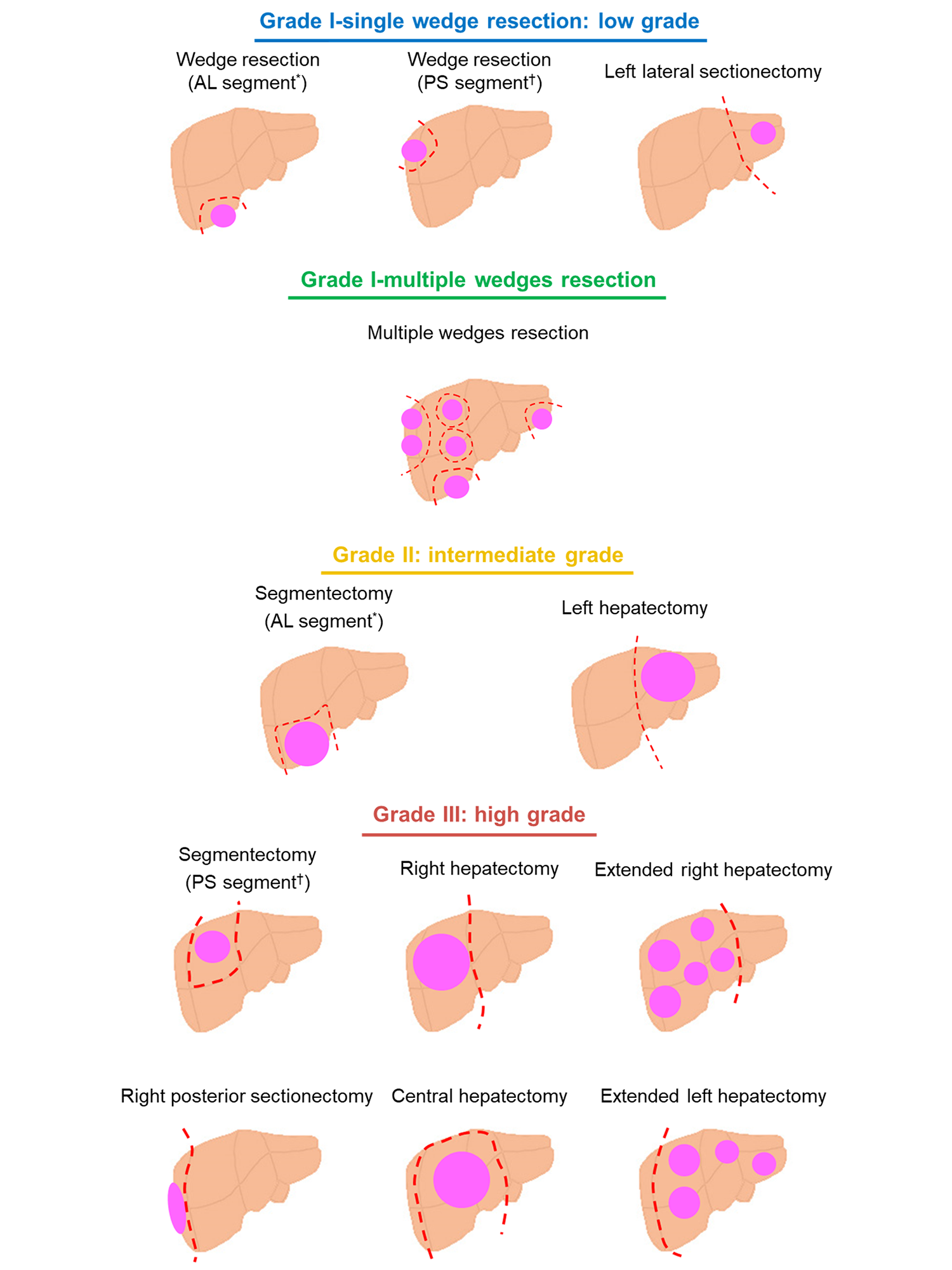

Supplement: Supplementary file 1 — Supplementary Figure 1. Modified complexity classification [from our previous work (22–24)] [file 10434_2025_17658_MOESM1_ESM.tif]
