# Supplementary material for: Novel Risk Score Incorporating Type-IV Collagen, Albumin, and Prothrombin Time (CAP score) to Predict 180-Day Surgery-Related Mortality After Liver Resection for Hepatocellular Carcinoma
Source: Ann Surg Oncol. 2025 Jun 23;32(10):7970–80. doi: 10.1245/s10434-025-17658-2 (PMC12454577; doi:10.1245/s10434-025-17658-2)
Supplement: Supplementary file 3 — Supplementary file3 (DOCX 16 kb) [file 10434_2025_17658_MOESM3_ESM.docx]

| **Supplementary Table 1. Detail of 180-day surgery-related death** | | |  |  |
| --- | --- | --- | --- | --- |
| Cause of death | N | survival date of all patients, day | 90-day mortality number, n (%) | 91-180 day mortality number, n (%) |
| Liver Failure | 34 | 17, 34, 34, 35, 40, 42, 43, 47, 50, 56, 58, 59, 62, 62, 63, 69, 69, 73, 73, 79, 88, 92, 100, 103, 131, 133, 134, 141, 141, 145, 149, 154, 164, 176 | 21 (61.8) | 13 (38.2) |
| Others |  |  |  |  |
| Pneumonia | 6 | 64, 87, 93, 101, 110, 138 | 2 (33.3) | 4 (66.7) |
| Sepsis | 5 | 72, 120, 140, 165, 168 | 1 (20.0) | 4 (80.0) |
| Multiple organ failure | 3 | 12, 22, 32 | 3 (100) | 0 (0) |
| General physical deterioration | 2 | 157, 180 | 0 (0) | 2 (100) |
| Cerebral stroke | 2 | 83, 151 | 1 (50.0) | 1 (50.0) |
| Gastrointestinal hemorrhage | 2 | 47, 144 | 1 (50.0) | 1 (50.0) |
| Renal failure | 2 | 60, 77 | 2 (100) | 0 (0) |
| Acute respiratory distress syndrome | 1 | 24 | 1 (100) | 0 (0) |
| Respiratory failure | 1 | 20 | 1 (100) | 0 (0) |
| Heart failure | 1 | 32 | 1 (100) | 0 (0) |
| Myocardial infarction | 1 | 48 | 1 (100) | 0 (0) |
| Total | 60 | - | 35 (58.3) | 25 (41.7) |

| **Supplementary Table 2. Uni- and multivariate analyses for 180-day surgery-related mortality using hyaluronic acid as fibrosis marker in training cohort (N = 623)** | | | | | | |
| --- | --- | --- | --- | --- | --- | --- |
| Factor | Univariate analysis | | | Multivariate analysis | | |
|  | OR | 95% CI | *P* | OR | 95% CI | *P* |
| **Liver function** |  |  |  |  |  |  |
| Albumin ≤3.4 g/dL | 6.63 | 2.97-16.8 | < 0.001 | 3.06 | 1.26-8.22 | 0.013 |
| PT-INR ≥1.26 | 5.09 | 2.26-10.8 | < 0.001 | 2.65 | 1.08-6.24 | 0.035 |
| Hyaluronic acid, ≥155 ng/mL | 7.83 | 3.18-23.6 | < 0.001 | 4.99 | 1.93-15.5 | < 0.001 |
| Abbreviations: OR, odds ratio; CI, confidence intervals; PT-INR, prothrombin time-international normalized ratio. | | | | | | |

| **Supplementary Table 3. Comparison of alternative Hyaluronic acid-Alb-PT-INR classification with existing models for predicting 180-day surgery-related mortality in total cohort** | | | |
| --- | --- | --- | --- |
| Models | 180-day mortality | | |
|  | AUC | *P*^*^ | |
| Hyaluronic acid–Alb–PT-INR classification | 0.721 | – | |
| MELD score | 0.557 | < 0.001 | |
| Child Pugh classification | 0.637 | 0.002 | |
| ALBI grade | 0.668 | 0.067 | |
| Abbreviations: ALBI, Albumin-Bilirubin; AUC, area under the curve; MELD, Model for End-Stage Liver Disease; PT-INR, prothrombin time-international normalized ratio | | | |
| *Versus Hyaluronic acid-Alb-PT-INR classification model | | |  |
